# Is the Planeterranean Diet the Key Driver Towards Health and Environment Promotion? A Narrative Review

**DOI:** 10.3390/foods14223920

**Published:** 2025-11-17

**Authors:** Milia Tzoutzou, Maria-Eleni Efthymiadou, Paraskevi Detopoulou, Tonia Vassilakou

**Affiliations:** 1 Department of Nutrition and Dietetic Sciences, Hellenic Mediterranean University, 72300 Siteia, Greece; miltzou@hmu.gr; 2 Department of Public Health Policy, University of West Attica, 11521 Athens, Greece; marialenae@gmaill.com; 3 Department of Nutritional Sciences and Dietetics, University of the Peloponnese, 24100 Kalamata, Greece; p.detopoulou@uop.gr

**Keywords:** Planeterranean diet, Mediterranean diet, plant-based diet, sustainable nutrition

## Abstract

This review describes the impact of current dietary patterns on health and food insecurity, emphasizing the need for a Planeterranean diet that boosts health and helps the environment. Current dietary patterns in most industrialized countries are both harmful for health and the environment and are connected to food waste despite existing policies to reduce it. Nutrition transition to Western-style diets, high in ultra-processed foods, increases environmental pressures and gas emissions. Sustainable diets promote biodiversity, reduce carbon footprints, support equity, and prioritize local, seasonal, organic, minimally processed, and culturally relevant foods. The Mediterranean diet and vegetarian diets constitute sustainable dietary patterns that benefit health and the planet. The “Planeterranean” perspective is a new scientific concept based on the Mediterranean diet, aiming to identify a sustainable food model based on locally available foods worldwide. The achievement of sustainable nutrition requires responsible agriculture, targeted policies, and community engagement. Universal guidelines must respect diverse cultures and regions, and a One Health approach is essential to improve well-being by ensuring food safety. This review critically evaluates the conceptual overlaps and limitations of these models and proposes directions for integrating sustainability and cultural aspects in policies and dietary recommendations.

## 1. Introduction

Our societies and healthcare systems are facing the increasing problem of abundance and food insecurity. Today, enough food is produced to ensure that a significant proportion of the population consumes approximately 2850 calories per day, yet we still cannot eliminate hunger [1]. More specifically, 1.9 billion people are overweight or obese, while nearly 800 million people are undernourished, and 2 billion people suffer from micronutrient deficiencies, known as “hidden hunger” [2]. The prevalence of food insecurity is also high since 45.3% of university students [3] and 50.4% of older participants [4] have been reported to experience severe food insecurity.

Key drivers of food insecurity include climate change, economic crises, conflicts, poverty, and the COVID-19 pandemic, which worsened existing inequalities [5]. People living in high-income countries in the Northern Hemisphere claim significantly more agricultural land than those in the Southern Hemisphere, as their diets rely heavily on animal products, which require much more land to produce than plant products [1]. The agricultural use of land for either plant crops or animal feed is related to low food production. Where these items are exported, food distribution is even more unfair. Second, insecure land-use rights, particularly land grabbing prevalent in low-income nations, significantly contribute to growing food insecurity [5,6]. High income inequality reduces the likelihood that food reaches the poorest segments of the population, even when food production and availability are sufficient [7]. These structural inequalities highlight the complex and multifaceted nature of food insecurity, which refers to a lack of consistent access to sufficient, safe, and nutritious food necessary for healthy growth, development, and an active life [8].

In this context, sustainability becomes the long-term component of all levels and dimensions of food security, the established and accepted determinant of a nation’s health and well-being [9]. In 1992, at the United Nations Conference on Environment and Development, the definition of “Sustainable Development” included three distinct dimensions: environment, economy, and society. When combined and put into practice, these three aspects can form a solid foundation for a sustainable world. The concept of “health” was included as a fourth dimension in the definition of sustainable development since the 1980s, given that nutrition has a significant impact on human health. “Culture” was then added as a fifth dimension, as cultural background influences dietary habits [10]. In the current sustainability goals of the United Nations, “zero hunger”, “good health”, “sustainable cities and communities”, “reduced inequalities”, and “responsible consumption and production” are present [11].

Sustainable diets have thus emerged as a challenging public health issue, as well as a critical matter for sustainable food systems at the core of global health, environmental, social, and economic challenges [9,12]. Global food production poses a significant environmental threat, and alongside agricultural technological advances, changing diets are suggested to mitigate the food system’s environmental impacts [13]. Indeed, high-income countries are urged to reduce emissions by incorporating renewable energy sources, improving efficiency, managing land sustainability, preventing deforestation, and encouraging climate-friendly lifestyles [14].

Diets that include plant-based foods and are low in meat, fish, and animal products have a lower environmental impact. As a result, governments, health councils, and nutritional institutes are incorporating sustainability into traditional dietary guidelines to promote diets beneficial for both health and the environment [10]. Among others, the Mediterranean diet (MD), based on the consumption of plant-based foods, has been characterized as a community-friendly, economical, and planet-friendly pattern [15]. However, the MD cannot be implemented globally due to variations in food production, availability, and cost. The “Planeterranean” perspective is a newly introduced concept that identifies healthy food models based on foods available in different areas of the world that are close to the nutritional value of foods of the MD [16]. Τhe planetary health diet, as defined by the EAT-Lancet Commission, is a flexible dietary framework. It provides recommendations per food category, which can be further tailored to fit local food availability, traditional cooking recipes, and preferences [17].

Differences in content or the extended dimensions of the Planeterranean diet and other sustainable diets (i.e., the MD, the wholesome diet/nutrition [10], or the vegan diet) and frameworks (planetary health diet) need to be better described. Thus, the aim of the present narrative review is to clarify newly arising definitions and further describe the impact of current dietary patterns on both health and food insecurity, emphasizing the need for a Planeterranean diet that boosts health and helps the environment.

## 2. Methods

Ιn terms of differentiating the Planeterranean diet concept from existing diets, clarifying its core characteristics, and providing examples of how this diet might be practiced in different regions, we conducted an advanced search of scientific databases based on specific keywords. In particular, a literature search was conducted across the electronic databases PubMed and Scopus, identifying studies published between 2000 and 2025. The selection of time frame was based on a preliminary exploration conducted by the authοrs, which revealed that most of the articles related to the research axis of sustainable diets were published after the year 2000. The following keywords and their combinations were used: “Planeterranean diet”, “Sustainable diet OR/AND nutrition”, “Plant-based diet”, and “Food security”.

Additionally, practical tips for sustainable everyday nutritional habits based on the Planeterranean diet were reviewed and presented herein. Inclusion criteria: (i) studies published in peer-reviewed scientific journals, either as original research, systematic reviews, or meta-analyses, and (ii) English or Greek language. Exclusion criteria: (i) published data in other languages. Gray literature, i.e., papers that were not peer-reviewed, such as those presented at conferences or included in dissertations, i.e., in Google Scholar or thesis projects, was not systematically reviewed. Due to the broad nature of the topic and the non-systematic bibliography search, a narrative approach was deemed more appropriate than a systematic review.

## 3. Results

### 3.1. The Principles of Sustainable Diets and Sustainable Development Goals (SDGs)

**Figure 1:**
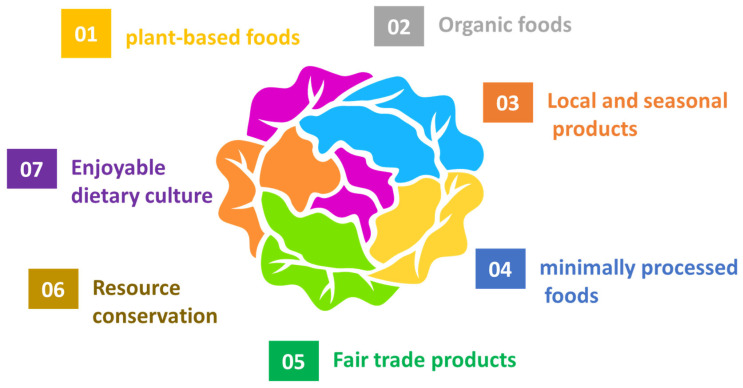
The seven principles of sustainable diet.

Over the last 40 years, the concept of the sustainable diet has been developed, characterized by the following seven principles: (1) preference for plant-based foods, (2) organic foods, (3) local and seasonal products, (4) preference for minimally processed foods, (5) fair trade products, (6) resource conservation, and (7) an enjoyable dietary culture that characterizes the so-called Wholesome Nutrition (Figure 1). These seven principles are interconnected with the five dimensions of sustainable development—environment, society, economy, health, and culture—and will be briefly analyzed below [10].

A critical review of the strengths and limitations of each sustainability principle is shown in Table 1.

#### 3.1.1. Preference for Plant-Based Foods

A preference for plant-based foods may reduce the consumption of animal products, offering ecological benefits such as lower greenhouse gas emissions and reduced water usage [18]. Plant-based foods require less land and resources than animal products, as converting plants into animal products is inefficient. This transition leads to a more sustainable production system [10]. Higher intake of complex carbohydrates, lower consumption of saturated fat, cholesterol, and purines are some of the potential health benefits. Research highlights that vegetarians exhibit better health outcomes compared to non-vegetarians, such as improved language [19] and memory functions [20]. In addition, plant-based diets are more cost-effective as the expenses for food purchases are decreasing [21]. Recently, a cross-sectional study among 398 elderly adults in Iran found that higher adherence to the Planetary Health Index (DPHI) is associated with reduced dietary costs [22], but no significant association was observed among single individuals or those with lower incomes. Lastly, vegetarians, vegans, or pescatarians avoid meat consumption as they are driven by moral considerations and taste preferences, and they align with a sustainable way of life [23]. A recent study in a Greek sample showed a positive association between a healthy diet, orientation, plant-based foods, and organic awareness [24]. However, according to earlier studies, many consumers tend to underestimate the environmental impact of meat consumption and show a clear unwillingness to pay higher amounts for meat alternatives. The global introduction of insects as a food source currently seems challenging [25].

#### 3.1.2. Organic Foods

Organic foods produce fewer harmful residues and offer environmentally friendly benefits like reduced soil desertification, enhanced biodiversity in ecosystems, and less hazardous residues. Organic food emits 40% less nitrous oxide than traditional farming practices [26]. Organic farming also promotes animal welfare and prevents the use of Genetically Modified Organisms (GMOs) and food irradiation [27]. Health-wise, organic foods may have higher nutritional value, although more data are needed [28]. They have lower levels of toxic metals, synthetic fertilizers, and pesticide residues, and can reduce exposure to antibiotic-resistant bacteria [29]. However, a large-scale shift to organic farming would probably necessitate converting more natural habitats into farmland. This expansion of agricultural land would lead to environmental impacts that are not fully captured when comparing results only on a per-unit-of-output basis. In addition, for some vegetable crops, organic farming can sometimes demand more fuel, especially when multiple rounds of mechanical or thermal weed control are needed [30].

#### 3.1.3. Local and Seasonal Products

Supporting small, local producers helps communities and strengthens the buyer-seller relationship and fair trade. The global food trade has increased the distance food travels, but seasonal products are those produced and consumed in the same climate zone, reducing energy use for refrigeration and heating. Local products result in lower carbon emissions, support small businesses, and may contain fewer harmful residues such as nitrates and pesticides [31]. Choosing to consume locally produced foods is linked with a reduced environmental footprint. A higher CO_2_ footprint is observed in Southeast Asia due to the adoption of an energy-intensive production process involving the use of heated greenhouses. Additionally, imported foods carry a higher CO_2_ footprint as they are transported via various means (by air, road, or sea), consuming significant amounts of energy. A portion of beef produces more greenhouse gas emissions compared to most other foods, while a chocolate bar originating from deforested tropical forests clearly has a greater ecological impact [32].

#### 3.1.4. Minimally and Ultra-Processed Foods

Minimally processed foods provide essential nutrients, offering humans a diet of higher nutritional density. Ultra-processed foods (UPFs) encompass ready-to-eat as well as ready-to-heat foods, produced through industrial methods. Some examples of UPFs are cereal bars, savory snacks, processed meats, pre-packaged frozen meals, soft drinks, sweetened beverages, instant soups, and sauces [33]. Over the last decade, studies have illustrated the potential harms associated with UPFs, which include an increased risk of non-communicable diseases, poor mental health, and early mortality [34]. More precisely, UPF consumption has been linked to several health complications, such as obesity, cardiometabolic diseases [35], and a higher risk of developing hypertension [36]. A recent study in a sample of university students showed that phase angle, which is an indicator of cellular health, was inversely related to UPF consumption and positively related to minimally processed foods consumption [37]. Furthermore, among children and adolescents, high UPF consumption was associated with increased levels of total and LDL cholesterol [38,39]. On the contrary, minimally processed foods have been linked to a high adherence to the MD [40]. It seems that foods that need to be consumed in moderation for health reasons are also the ones that have the most significant negative impact on the environment. Moreover, energy-intensive food processing also contributes to pollution and water waste [35]. Considering that food waste is quickly becoming a significant global issue due to its environmental impacts, studies have shown that UPF consumption plays a role in increasing food waste. For example, in Denmark, processed foods are more frequently wasted than unprocessed foods [41], while in the UK, unprocessed foods are those mostly wasted [42]. The energy-intensive production of UPFs, combined with the fact that many of them are wasted, does not support the sustainability concept.

#### 3.1.5. Fair Trade Products

Fair trade ensures higher incomes for producers in low-income countries by offering fair prices and long-term contracts, excluding child and forced labor. Also, in high-income countries, fair prices contribute to the livelihood of small producers and create new workplaces in rural areas. The global concentration process of big companies in farming, processing, and retailing is a huge problem for small and medium-sized enterprises, because they cannot compete with the low prices of big companies. It also promotes environmental sustainability, using fewer chemicals, offering drinking water protection, promoting reforestation, and encouraging organic practices [43]. Higher wages improve local health, education, and food security. It is also important to check the labelling of packaged foods and look for certifications or quality seals on the packaging (such as RSPO—Roundtable on Sustainable Palm Oil) or ASC (Aquaculture Stewardship Council) for responsible aquaculture [44].

#### 3.1.6. Resource Conservation

The concept of resource conservation centers on the ethical and efficient utilization of natural resources, aiming to secure their long-term supply while minimizing ecological harm [45]. It requires optimizing resource use, minimizing waste creation, and protecting ecological systems. A balance between human needs and environmental protection, assuring that natural resources are used efficiently for current and future generations, is crucially needed [45]. By conserving resources like water, energy, minerals, and biodiversity, we can lessen resource depletion, minimize pollution, and promote ecological balance, thereby fostering a more sustainable and resilient world environment. Utilizing energy-saving technologies can decrease greenhouse gas emissions, significantly aiding efforts to combat climate change [46]. Organic farming and agroforestry are considered sustainable agricultural methods that enhance biodiversity while fostering ecosystem adaptability. For example, the use of traditional agricultural technologies might decrease the land available for pork breeding within the EU by 20% [47]. In order to manage household sustainability, people should ideally adopt a sustainable lifestyle, such as transitioning to renewable energy resources, utilizing energy-efficient appliances, choosing alternative ways of transportation—such as walking or biking—and minimizing food waste, given that this constitutes a large part of total food waste [48].

#### 3.1.7. Enjoyable Food Culture

Food and culture are deeply connected. Simple food practices like preparation, serving, and sharing also hold significant social and cultural meaning. Culinary traditions and recipes serve as a means of intergenerational knowledge transfer [49,50]. Importantly, enjoying food is not at odds with achieving health, ecological, economic, and social sustainability; in fact, it is crucial for long-term sustainable food systems, as delicious meals can align with these requirements. Interestingly, in the updated pictorial representation of the MD, “conviviality” and “eating together around the table” are central features [51].

#### 3.1.8. Sustainable Diets as Part of Sustainable Development Goals

Sustainable diets are in line with the Sustainable Development Goals (SDGs) and aim to protect and respect biodiversity and ecosystems. They are culturally acceptable, affordable, economically fair, nutritionally adequate, safe, and healthy, while optimizing natural and human resources [52]. The SDGs can address food consumption with a lower water and carbon footprint and promote the use of food biodiversity, including traditional as well as seasonally and locally produced foods, with nutrient-rich species and varieties [14,53]. Additionally, a sustainable diet can help reduce greenhouse gas emissions in the food sector. Adopting a diet aligned with this concept can ultimately contribute to limiting global warming and to protecting against the climate crisis [10]. In addition, it preserves the environment, biodiversity, and local resources [12]. Interestingly, a systematic review of diet modelling scenarios found that sustainable diets can lead to gas emission reductions of more than 20% [54].

#### 3.1.9. Sustainable Diets and Nutritional Status

As research focuses more on sustainable diets, it is crucial to look at both their environmental benefits and how well they meet people’s nutritional needs. Well-designed sustainable diets include vegetables, fruits, whole grains, moderate dairy, fish, and poultry, and limit sweets and red meat. These changes potentially improve nutrient quality. However, these diets may compromise the intake of certain micronutrients, such as vitamin B12, iron, zinc, calcium, and vitamin D. Moreover, environmental changes such as increasing atmospheric CO_2_ levels may further affect the nutritional quality of food crops. Myers et al. [55] report that, given that global [CO_2_] is expected to reach 550 ppm in the next 40–60 years, the edible parts of many major crops (i.e., rice, potato, starch, maize) show reduced nutritional value (especially in dietary zinc, iron, and protein) compared to plants grown under current [CO_2_] levels. This is an important global issue related to sustainable food security and adequacy.

Payne et al. [56] systematically reviewed the results of published studies that link greenhouse gas emissions (GHGEs) of dietary patterns to nutritional content or associated consequences for health. The outcomes exhibited significant heterogeneity. Results linking reduced GHGEs to lower levels of nutrients were inconsistent. Most diets with lower GHGEs showed reduced salt and saturated fat, likely due to reduced meat intake. Eight of twelve studies analyzing salt and fat included diets with less meat and dairy. However, sugar intake increased in most low-GHGE diets, for unclear reasons. In Denmark, Lassen et al. [57], based on the EAT-Lancet reference diet, developed a healthy plant-based diet that aligned with the national dietary guidelines, also considering local food culture and availability. Firstly, the researchers developed Model 1, based on the EAT-Lancet diet and on food availability in Denmark. Then, it was adapted to reflect national dietary guidelines and consumption habits, including processed foods and beverages. Results showed significantly lower amounts of whole grains, legumes, and vegetable fats in Model 2 compared to Model 1, while notably higher quantities of potatoes, fruits and berries, dairy products, and fish were observed in Model 2. The diet’s content of macronutrients, as well as vitamins and minerals—except for vitamin D and iodine, nutrients already lacking in the typical Danish diet—was found to be adequate according to the Nordic Nutrition Recommendations (NNR) for individuals aged 6–65 years.

A recent systematic review tried to evaluate the possible effects on micronutrient intake due to the transition to a diet aimed at diminishing environmental impact. In most of the studies examined, it was found that micronutrients such as vitamins A, D, B12, and folate would decrease, while calcium, iron, iodine, and zinc would increase. However, the review found no observational data on micronutrient status; all of the 55 observational studies reported dietary intake data and were secondary analyses, with wide variations in study design, sample size, dietary assessment methods, and data reporting. Researchers underline that in order to understand the impact of sustainable diets on micronutrients, well-designed intervention studies and standardized observational data, including biomarkers, are needed [58]. Contrasting this, a US-based study that attempted to match diets to environmental impact data examined the relationship between self-selected diets and GHGEs. Low-GHGE diets were found to contain more fiber and vitamin E, and less sodium and saturated fats. However, they contained significantly less iron, calcium, vitamin D, vitamin A, choline, and potassium compared to high-GHGE diets. This is likely due to the lower consumption of animal foods, meats, and dairy, indicating the need for a balanced dietary guidance [59].

In the Malmö Diet and Cancer study (*n* = 25,970), participants consuming more climate-friendly diets had lower intakes of micronutrients and animal-sourced foods. However, they did not have an increased risk of deficiencies, highlighting the importance of assessing both dietary intake and biomarkers when evaluating sustainable diets [60]. To our knowledge, there is only one clinical trial investigating the effects of a sustainable diet on nutrient status. In this trial, the sustainable MyPlanetDiet led to healthier diets and lower diet-related gas emissions [61]. Other potential benefits that should also be mentioned include psychological and physical well-being, humane treatment of animals, food ethics, cultural and social diversity, and the exchange of knowledge [62,63].

### 3.2. Conceptual Comparison of Western Diets, Mediterranean, Planeterranean Diet, Planetary Health, and One Health

#### 3.2.1. Western Diets and Nutrition Transition

Western diets are shifting toward higher consumption of refined vegetable oils, refined sugars [7], and ultra-processed foods [64]. These changes require more land use since processed food production is associated with intensive agriculture and livestock [65] and increased (GHGE) [66]. Vega et al. [67] examined the links between health, agricultural production, and environmental data, and showed that Western diets have negative consequences for all the aforementioned factors. Human diets cause a severe increase in GHGE production, and an expansion of land-use change to satisfy the demand to produce highly processed foods and sugary drinks. Quantitative assessments further highlight the magnitude of this impact. In quantitative terms, the estimated daily carbon footprint from the Western diet ranges between 5.5 and 23.2 kg CO_2_e/day/person, mainly due to high consumption of red meat, but also of processed foods, refined grains, added sugars, and animal-based fats. In contrast, the Mediterranean diet results in 0.7–7.13 kg CO_2_e/day/person, driven by higher consumption of whole grains, fruits, vegetables, legumes, nuts, and olive oil, moderate fish/poultry, and low consumption of red meat and processed foods [68,69,70].

However, the effects of dietary shifts or dietary differentiations might vary based on existing consumption patterns according to the specific characteristics of each country (e.g., location) under examination. To ensure that humans live within the global food carbon budget, it is necessary to address the imbalance in global food-related emissions. For example, Sweden’s per capita food-related GHG emissions—whether compared to the current level or aligned with NDGs—are more than double those of Malawi, even though Malawi still faces serious malnutrition problems. Western countries such as Australia, Argentina, Brazil, and France would need to achieve substantially greater reductions in food-related emissions, whereas countries such as Bangladesh, Indonesia, and Ethiopia, with more flexitarian-oriented dietary patterns, would better meet sustainability goals [71]. Another systematic review revealed that transitioning from typical Western diets to more environmentally sustainable eating patterns could lead to reductions exceeding 70% in GHGE and land use, and up to 50% in water use [54]. Adopting a Western diet in low-income countries can thus increase land use requirements [72]. At the same time, the demand for meat and milk is increasing among the emerging middle class in developing countries [73], while sales of ultra-processed foods are growing rapidly in Asia, the Middle East, and Africa [64]. Even India, a country known for being primarily vegetarian, is recording increasing demand for meat, driven by its growing middle class. As a result, these dietary changes are expected to lead to much higher land use due to population growth [74].

#### 3.2.2. Mediterranean Diet

As public dialogue continues at a global level to find solutions for more sustainable food systems, interest in the MD has increased. The MD is well known to be beneficial for both humans and the environment. The Food and Agricultural Organization (FAO) has recognized it as an exemplary sustainable diet, valued for its nutritional value since the mid-20th century [14], and UNESCO designated it as an intangible cultural heritage of humanity [75].

The MD has a smaller ecological footprint than typical diets in industrialized nations, particularly the Western dietary pattern model, due to its emphasis on local, seasonal plant-based foods and reduced animal product intake [76]. The health benefits of the MD were first documented in the Seven Countries Study in the early 1960s [77]. Extensive research since then shows that the MD offers protective benefits against cardiovascular disease, chronic and degenerative conditions [78], and type 2 diabetes, while promoting longevity and improving quality of life [79]. Higher MD adherence has been linked to a favorable body composition profile [80] and potential protection against COVID-19 [81]. In addition, those adhering closely to the MD also meet their micronutrient needs more effectively than those on a standard Western diet [82].

In 2011, the FAO and the International Centre for Advanced Mediterranean Agronomic Studies (CIHEAM) collaborated to assess the MD as a model for sustainable food systems, developing indicators and a framework through international workshops. In 2015, the Med Diet 4.0 framework emerged, highlighting the MD’s multiple sustainability benefits: (1) health benefits for chronic disease prevention and well-being, (2) low environmental impact and biodiversity support, (3) positive local economic impact and waste reduction, and (4) social and cultural value through identity and inclusion [71]. The International Foundation of Mediterranean Diet (IFMeD) furthered this consultation to update the MD pyramid, integrating recent scientific insights that underscore the MD’s sustainability and health benefits. The innovation of the updated MD lies in its third dimension, representing the environmental impact of included foods and aspects of the sustainability of food production. Therefore, the MD should be viewed as it is: an exceptionally and incomparably healthy, affordable, and environmentally sustainable dietary model, as well as an ancient cultural heritage that provides identity and belonging [71]. The MD is not only a model of cultural food choices, cooking methods, meal patterns, and a broader lifestyle, but also a sustainable framework that alleviates the environmental pressure of food production and consumption. Broader adherence to this dietary model would contribute significantly to greater sustainability of the food system, with numerous benefits for human and planetary well-being [83].

At the same time, the Double Pyramid Model illustrates that foods recommended for frequent consumption generally have a lower environmental impact, while those advised in moderation have higher impacts. This model links the nutritional value of foods with their environmental costs, showing that items advised to be of limited consumption for health reasons often also have greater land, water, and CO_2_ footprints [14]. A comparison of three nutritionally balanced menus, differing only in the proportion of animal products included, found that a diet based on MD principles, as outlined by the Double Pyramid, has a lower environmental impact than diets with high daily meat consumption [14]. Moreover, in the updated pictorial scheme of the MD by the Italian Society of Human Nutrition, the sustainability and biodiversity principles are clearly shown at its basis [51]. According to a recent meta-analysis, the MD was overwhelmingly identified (91% of studies) as a sustainable dietary pattern that aligns with planetary health. Specifically, the MD’s environmental footprints were reported as ranging from 1.03 to 5.08 kg of CO_2_ equivalent per person per day for greenhouse gas emissions, 257.2 to 2735.2 L per person per day for water footprint, and 4 to 14.8 square meters per person per day and 2.85 to 3.32 square meter-years per day for land use [15].

Vassilakou et al. created a guide for young people’s nutrition using mainly plant-based Greek ingredients, promoting plant-based eating with a lower environmental impact. Practical guidelines were given to achieve optimum intake of essential minerals and nutrients. For example, 50 g of dried figs provides the same calcium as 100 g of cow’s milk, and 90 g of walnuts or 80 g of oats match the protein of two slices of ham. Basic info on reducing gas emissions by promoting plant-based foods was also provided. A snack of apples with cinnamon, peanut butter, and honey generates 0.14 kg CO_2_-eq, while a spinach trahana pie produces 0.3 kg CO_2_-eq [84].

#### 3.2.3. Planeterranean Diet (PD)

Colao et al. 2022 [85], on behalf of the UNESCO Chair on Health Education and Sustainable Development, tried to expand the adjustment of a dietary pattern close to the MD in non-Mediterranean countries, i.e., countries in Asia. Indeed, the Planeterranean diet takes the core principles of the Mediterranean diet, such as high consumption of fruits, vegetables, whole grains, legumes, nuts, olive oil, and moderate intake of fish and dairy, and adapts them to local ecosystems and cultures around the world. Its goal is to make the Mediterranean diet’s health and environmental benefits globally accessible by encouraging the use of locally produced, seasonal, and culturally relevant foods that follow similar nutritional and sustainability principles. For example, substituting olive oil with avocado in Central America or marine algae for certain nuts in Asia, and eating according to a dietary pattern that focuses on locally grown vegetables, legumes, and fish in Japan, and beans, quinoa, and native fruits in South America.

Implementing nutritionally sustainable diets requires practical guidance and balance to ensure consumers have both knowledge and access to appropriate foods. If food-related GHG emissions were reduced by 90%, the respective dietary pattern would include just seven plant-based foods. In addition to the restrictive and poor variety of this diet, additional problems would arise concerning the unfeasibly large quantities of food that humans would need to consume in order to meet their nutritional requirements. Το overcome this challenge, Macdiarmid et al. proposed the adoption of a dietary pattern including fifty-two foods, primarily plant-based, along with moderate consumption of dairy products, fish, and limited intake of red meat [86]. This diet adequately meets nutritional requirements with realistic food quantities and variety. Recommended foods were listed in grams of consumption on a weekly basis, and as expected, plant-based foods dominated. Given the potential challenges in meeting the requirements for protein, iron, and calcium on a plant-based diet, it is advisable to wisely calculate their intake [86].

#### 3.2.4. Planetary Health

The EAT-Lancet Commission on “Healthy Diets from Sustainable Food Systems” introduced the planetary health diet, a model for healthy and sustainable eating designed to promote both human and environmental well-being [17]. Cacau et al. [87], building upon the philosophy of the EAT-Lancet Commission project, proposed the development of the Planetary Health Diet Index (PHDI). Using nutritional data from the Longitudinal Study on Adult Health (ELSA), which took place in Brazil, the authors developed the PHDI by creating an updated tool combining factors such as food density, adherence in different caloric scenarios, and the association between food quality and environmental impact. The final index score showed a negative association with animal protein, total fat, saturated fat, and cholesterol, as excessive consumption of foods high in these is associated with adverse health and environmental outcomes. In October 2025, the EAT-Lancet Commission released an updated report emphasizing the urgency of transforming global food systems to sustainably feed nearly 10 billion people by 2050. It advocates for dietary shifts towards plant-based foods and systemic changes in agriculture and food distribution to achieve health and environmental goals [88].

#### 3.2.5. One Health and Sustainability

One Health is defined by the One Health High Level Expert Panel as an integrated, unifying approach that aims to sustainably balance and optimize the health of people, animals, and ecosystems [89]. The One Health approach highlights the crucial interconnection between the health of people, animals, and our planet [90]. Population growth and expansion have led to an increase in contacts between humans, humans and wildlife, as well as domestic animals and wildlife. These developments are reinforced by other factors, such as climate change, deforestation, and intensive farming, which destroy wild animals’ habitats. The movement of people, animals, and animal products, increased by globalization, contributes to the start, and spread of zoonoses [89]. For example, zoonotic diseases, such as COVID-19 and Ebola, demonstrate how pathogens circulating in animal populations can easily affect human health. Areas with frequent land-use changes and high pressure on biodiversity are identified as hotspots for the emergence of infectious diseases. Furthermore, climate change may exacerbate this process, leading to increasingly complex consequences [91]. Integrating animal health surveillance, environmental monitoring, and human health systems allows for early detection and prevention of such diseases. However, the implementation of One Health extends far beyond the traditional focus on Antimicrobial Resistance (AMR) and Zoonoses Control [89].

**Figure 2:**
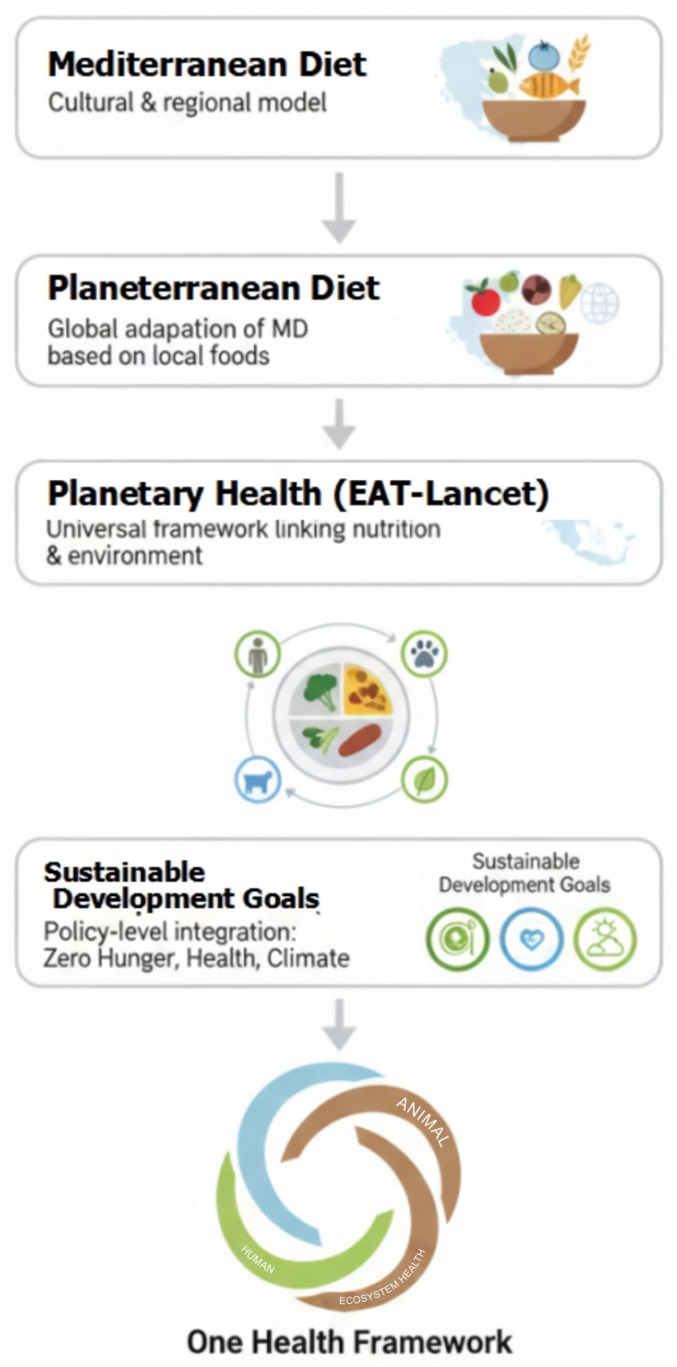
The concepts of Mediterranean diet, Planeterranean diet, Planetary health, and One Health framework.

The One Health framework supports sustainable food systems since it links agriculture, nutrition, and environmental protection. Practices such as responsible livestock management, reduction in antibiotic use, and promotion of plant-based or climate-friendly diets contribute to healthier animals, lower environmental impacts, and better nutrition. By addressing issues like food safety, antimicrobial resistance, and biodiversity loss collectively, One Health is not just a concept, but a multisectoral strategy where scientific, social, and governmental bodies must integrate their knowledge and resources to tackle health threats at the human-animal-environment interface. One Health provides a holistic strategy for meeting the challenges of global food security, climate change, and public health simultaneously. To meet the demands of global food security, protect our planet’s resources, and improve well-being by ensuring food safety, a One Health approach is essential [90]. The concepts of the MD, PD, planetary health, and One Health are depicted in Figure 2.

## 4. Policies for the Environment, Food, and Health

It seems more crucial than ever to promote commitments to sustainable development, something that can be achieved through consumer education and sustainable diets, as an alternative way of life. It is necessary to increase our appreciation for food. To achieve this goal, scientists, stakeholders, and consumers must support “Education for Sustainable Development.” This is one of the new SDGs set by the United Nations in September 2015, aiming at driving the shift toward a more sustainable society and lifestyle [10].

While there is growing scientific evidence on the environmental impact of diets and their connection to public health and sustainability, there is a slow incorporation of sustainability features into dietary guidelines. Several countries, including Qatar, Brazil, Sweden, and the Netherlands, have integrated sustainability into their national dietary recommendations. The German Council for Sustainable Development has created a “basket of sustainable shopping” to guide consumers toward sustainable food purchases. In addition, many challenges remain in understanding the complexities of sustainable diets, assessments, and determining factors [9]. However, the implementation of sustainable nutrition policies may deal with several challenges and obstacles, including political and economic obstacles (e.g., industry lobbying, conflicting policy mandates, trade policies and subsidies, political timelines), logistical challenges in terms of supply chain and infrastructures (e.g., supply chain inertia, producer risk and transition, prices and affordability, food services/institutional procurement) and socio-cultural and behavioral obstacles (e.g., cultural significance of red meat and dairy, skill deficits and culinary literacy, perception of taste, satiety and convenience, misinformation and consumer confusion) [92].

Current food consumption in industrialized countries harms both health and the environment. Raising awareness about the environmental and nutritional impacts of food choices is essential, allowing individuals and organizations to collaborate effectively and improve global nutrition [93]. People who eat locally, seasonally, organically, or adopt a vegetarian diet have a lower environmental impact than those on conventional diets. Thus, transitioning to sustainable diets focused on organic, local, and seasonal foods supports sustainable development goals [31]. It is important to promote the MD as a sustainable lifestyle rooted in the cultural identities of Mediterranean peoples. This diet is better suited to modern times, diverse population groups, and various life stages, while incorporating sustainability into contemporary food culture [9]. In addition, sustainable nutrition for all requires the responsible development and use of new agricultural technologies. These technologies and policies should prioritize nutritional diversity and minimize environmental impacts [2].

One Health is an integrated framework for achieving sustainable health and security across diverse environmental, agricultural, and human health domains, rather than just a tool for fighting existing diseases. In this context, the European Commission has launched significant policy initiatives such as the “From farm to fork” strategy, several relevant regulations, the European Health Emergency Preparedness and Response Authority (HERA), and the Global Health Strategy [90].

According to the revised report of EAT (in 2025), policy changes such as taxing unhealthy foods, subsidizing nutritious options, regulating harmful food advertising, and improving labor conditions in agriculture are crucial [94].

The introduction of an integrated sustainability framework in schools and among health professionals can be a significant step toward a broader application of sustainability principles. A recent review of school-based nutrition programs revealed key factors affecting their long-term success [95]. The primary obstacle identified was insufficient organizational preparedness and resources, while strong external partnerships and a positive surrounding context were the most frequent facilitators [89]. In addition, health professionals are well-informed and have a positive attitude towards sustainable diets [96].

## 5. Discussion

In this narrative review, sustainability concepts of diets are presented with an emphasis on the MD and the PD. The PD is an innovative concept, but currently relies mostly on empirical research. Indeed, many of the benefits of the PD are based on extrapolations from the well-documented Mediterranean diet rather than from studies specifically assessing Planeterranean adherence, nutrient adequacy, or sustainability outcomes. To date, there are no standardized indices to quantify adherence to the PD in different regions, so future research should focus on developing measurable parameters, such as greenhouse gas emissions per kilocalorie or nutrient density scores, tailored for each region.

Another challenge lies in the potential cultural generalization of Mediterranean culture. The PD aims to globally adapt Mediterranean principles by identifying local foods with similar nutritional and environmental profiles. However, such transposition may result in a simplification of traditional dietary systems. True sustainability requires respect for local food heritage, culinary traditions, and socioeconomic conditions. A culturally adaptive approach, grounded in participatory research with local communities, dietitians, and agricultural stakeholders, should guide the translation of Mediterranean principles into regional dietary guidelines. In doing so, the Planeterranean concept could strengthen rather than replace existing cultural food practices. Moreover, significantly reducing meat consumption in societies where it is the norm presents challenges. Research is needed to understand the cultural and socio-economic factors influencing dietary changes [97].

Indeed, socioeconomic status and food accessibility influence the type and quality of nutrients available in food markets. These choices impact nutritional outcomes, with consumers’ ability to make such choices linked to income and food availability [98]. Sustainable living should be viewed as a healthy lifestyle pattern and promoted by health professionals through public health initiatives [99]. Current food consumption patterns in industrialized countries have negative impacts on both human health and the environment.

The implementation of refined policies towards the Planeterranean diet should target all sources of political conflict and misaligned mandates (e.g., by establishing independent dietary guidelines bodies), logistical obstacles (e.g., by implementing differential taxation/subsidy schemes, investing in decentralized infrastructure, developing comprehensive transition), and socio-cultural and behavioral challenges (e.g., by mandating practical food literacy, leveraging public procurement standards, funding national diet-climate campaigns) [92].

At the same time, food waste is rapidly emerging as a major global challenge because of its economic, social, and environmental impacts. One of the sustainable development goals of the United Nations was to reduce food waste by half by 2023 (SDG 12.3—Responsible Consumption and Production). Despite efforts to address environmental issues, in 2022, global food waste reached an alarming 1.05 billion metric tons, with only 9/193 countries including specific food waste reduction measures in their climate action plans (The Sustainable Development Goals Report 2024). In this context, it is important to raise public awareness about the environmental and nutritional implications of our dietary choices. In other words, individuals and organizations can effectively collaborate to accelerate improvements in global nutrition [91]. Individuals who choose local, seasonal, or organic foods and follow a vegetarian diet have a lower environmental impact than those on conventional diets. Transitioning to sustainable diets based on these principles presents an opportunity to promote sustainable development [31]. It is also essential to reinforce the MD as a sustainable lifestyle that reflects the cultural identities of Mediterranean people, adapting it to modern times and diverse populations while incorporating sustainability [9]. In addition, sustainable nutrition for all will not be possible without the responsible development and use of new agricultural technologies. These technologies and policies must prioritize nutritional diversity and reduce environmental impacts [2]. The ongoing crisis demands a politically correct and holistic transition from globalization to global health, as emphasized even before the spread of SARS-CoV-2. Stronger policies, programs, and research related to nutrition need to be developed, tailored to local, regional, national, or international contexts [91]. While all frameworks promote sustainability, the planetary health diet prioritizes global nutrient ranges, whereas the Mediterranean diet emphasizes cultural identity and local food systems.

The present work has several limitations. The literature review may not have captured all available studies examining Planeterranean nutrition and its benefits for humans, since it is not a systematic review, and furthermore, the gray literature in this field is vast. As a result, inter-regional or cross-country variations in the implementation or adaptation of sustainable nutrition may not be fully described here. In addition, the studies included herein encompass a diverse range of study designs and employ various indicators to address the issue of sustainable nutrition.

## 6. Conclusions

Today, the challenge of adapting to an environmentally friendly diet suitable for all cultures, populations, and geographic regions is evident. To address this, there is a need for the development of sustainable and healthy dietary recommendations that are tailored to specific regions and cultures, while also considering all aspects of sustainability. The Planeterranean diet emerges as a crucial necessity in the face of two converging global crises: the escalating epidemic of diet-related chronic diseases and the overwhelming environmental pressure from industrial food systems. It is essentially an adaptation of the highly acclaimed Mediterranean diet, recognized for its plant-rich, healthy principles, but with a critical update for global implementation. Its necessity lies in providing a scalable solution that maintains the proven health benefits while simultaneously aligning with the urgent need for environmental sustainability. This model moves away from shipping traditional Mediterranean staples worldwide, instead promoting the concept of a plant-based, seasonal, and minimally processed diet, using locally and traditionally available ingredients, thereby reducing transport emissions and supporting local biodiversity.

The necessity of the Planeterranean approach is fundamentally also about ensuring food security and ecological resilience in a climate-challenged world. Advocating for the use of locally adapted foods lowers the carbon and water footprint associated with globalized food supply chains. This shift decentralizes the food model, making healthy eating accessible and culturally relevant regardless of geography. Ultimately, the Planeterranean diet provides a pragmatic framework for policymakers, farmers, and consumers alike to transition towards a sustainable, lower-impact food system, addressing both human health and the planet’s ecological boundaries simultaneously. Furthermore, the partnership between nutrition professionals and community organizations creates a holistic approach to sustainable diets, while food waste reduction fosters sustainability and long-term changes. In addition, the involvement of various target groups in any future actions (public, educators, students, health professionals) serves as an amplifying sustainability factor.

More research on the Planeterranean diet is necessary because it is a new, conceptual adaptation that requires empirical validation to prove its intended benefits outside the Mediterranean basin. There is a critical need for randomized controlled trials (RCTs) and large-scale cohort studies to definitively demonstrate that adherence to these adapted, region-specific “nutritional guidelines” yields similar reductions in non-communicable diseases across diverse global populations (e.g., in Asia, Africa, or North America). Furthermore, extensive research is crucial to fully map the environmental and socio-cultural dimensions of the Planeterranean concept.

## Figures and Tables

**Table 1:** Critical appraisal of the seven principles of sustainable diets.

Principle	Core Idea	Strengths	Limitations	Suggestions
1. Plant-based foods	Based on fruits, vegetables, legumes, nuts, and whole grains	Reduces greenhouse gas emissions and resource use; improves cardiovascular and metabolic health; aligns with EAT-Lancet guidance	Potential low protein quality and micronutrient deficiencies (iron, B12, zinc); not all plant crops are low-impact (e.g., almonds, soy)	Include diversity of plant sources to achieve maximum benefits
2. Organic foods	Favor organic production to reduce chemical inputs and preserve ecosystems	Reduces pesticide exposure, supports biodiversity, and promotes animal welfare	Lower yields may cause land expansion; sometimes higher energy use for weed control; limited affordability	Combine different sustainable practices, focus on circular resource use
3. Local and seasonal products	Prefer foods produced close to consumption site and in season	Supports local economies, reduces transport emissions, and fosters cultural identity	All “local” foods are not necessarily sustainable	Balance local sourcing with global ethical trade
4. Minimally processed foods	Limit ultra-processed foods; choose fresh or lightly processed options	Strong epidemiological link between ultra-processed foods intake and chronic diseases; lower environmental impact from less energy-intensive processing	Some processing ensures safety and fortification; ignores role of innovation (e.g., fermentation, biofortification)	Differentiate between beneficial and harmful processing encourage reformulation
5. Fair trade products	Support equitable supply chains and fair compensation for producers	Promotes social justice, community stability, and ethical consumption	Certification coverage remains limited; high verification costs; not always linked to environmental outcomes	Integrate fair trade with sustainability certification and local coops
6. Resource conservation	Efficient use of water, land, and energy; reduction in waste	Encourages circular economy principles and ecosystem preservation	Lacks dietary application metrics	Link resource conservation to dietary indicators (e.g., water footprint per kcal)
7. Enjoyable food culture	Food as social, cultural, and sensory experience	Encourages long-term adherence, cultural continuity, and well-being	Overlooked in most dietary guidelines; Planeterranean adaptation risks cultural heritage	Highlight enjoyment and tradition as sustainability components

## Data Availability

No new data were created or analyzed in this study. Data sharing is not applicable to this article.

## Abbreviations

The following abbreviations are used in this manuscript:

ASC	Aquaculture Stewardship Council
MD	Mediterranean Diet
RSPO	Roundtable on Sustainable Palm Oil
UPF	Ultra Processed foods
